# A magnetoelastic biosensor based on E2 glycoprotein for wireless detection of classical swine fever virus E2 antibody

**DOI:** 10.1038/s41598-017-15908-2

**Published:** 2017-11-15

**Authors:** Xing Guo, Shengbo Sang, Jinyu Guo, Aoqun Jian, Qianqian Duan, Jianlong Ji, Qiang Zhang, Wendong Zhang

**Affiliations:** MicroNano System Research Center, Key Lab of Advanced Transducers and Intelligent Control System of the Ministry of Education & College of Information Engineering, Taiyuan University of Technology, Jinzhong, 030600 China

## Abstract

A wireless magnetoelastic (ME) biosensor immobilized with E2 glycoprotein was first developed to detect classical swine fever virus (CSFV) E2 antibody. The detection principle is that a sandwich complex of CSFV E2 – rabbit anti-CSFV E2 antibody – alkaline phosphatase (AP) conjugated goat anti-rabbit IgG formed on the ME sensor surface, with biocatalytic precipitation used to amplify the mass change of antigen–antibody specific binding reaction, induces a significant change in resonance frequency of the biosensor. Due to its magnetostrictive feature, the resonance vibrations and resonance frequency can be actuated and wirelessly monitored through magnetic fields. The experimental results show that resonance frequency shift increases with the augmentation of the CSFV E2 antibody concentration. Scanning electron microscopy (SEM), energy-dispersive spectroscopy (EDS) and fluorescence microscopy analysis proved that the modification and detection process were successful. The biosensor shows a linear response to the logarithm of CSFV E2 antibody concentrations ranging from 5 ng/mL to 10 μg/mL, with a detection limit (LOD) of 2.466 ng/mL and the sensitivity of 56.2 Hz/μg·mL^−1^. The study provides a low-cost yet highly-sensitive and wireless method for selective detection of CSFV E2 antibody.

## Introduction

Classical swine fever (CSF), induced by classical swine fever virus (CSFV), is a lethal and highly contagious disease which has a tremendous economic impact on the swine industry worldwide^1,2^. Some countries, such as Australia, North America, and New Zealand have successfully eradicated the disease through the fulfillment of regulatory measures^3^. However, the disease is still existent in other parts of the world, for instance, Madagascar, Singapore, Laos, Lithua-nia, Myanmar, Colombia, and Republic of Korea, impeding the development of animal husbandry^4–6^.

CSFV is an enveloped positive-stranded RNA virus in the Flaviviridae family under the genus Pestivirus, with a genome size of 12.3 kb and comprises of a single large open reading frame coding for a polyprotein of 3898 amino acids^7–9^. The polyprotein is processed into four structural proteins (C, E^rns^, E1, E2) and some nonstructural proteins by the cellular and viral proteases^10^. E2 is an envelope glycoprotein present on the surface of the virion and is the major target to induce protective immune response against CSFV infection in pigs^11,12^.

Therefore, CSFV E2 antibody detection is critical for diagnosis of CSF and efficient monitoring of vaccination in the CSF eradication work. Sensitive detection of CSFV E2 antibody is pivotal for prevention and control of CSF^13^. Various methods have been developed to detect CSFV E2 antibody, such as single dilution immunoassay^14^, indirect ELISA^15^ and surface plasmon resonance (SPR)^16^. However these methods have some limitations, such as work-intensive, time-consuming and high-cost. So a highly sensitive, inexpensive and facile method is necessary for the detection of CSFV E2 antibody.

In recent years, a thick-film mass-sensitive magnetoelastic (ME) sensor made of ferromagnetic metallic glass ribbons, such as Metglas 2826MB have gained considerable attention due to their remarkable features of low cost, ease of use, high sensitivity as well as wireless sensing^17–19^. In response to the superposition of both alternating (AC) and static (DC) magnetic fields, the ME sensor longitudinally vibrates at its resonance frequency^20^. As the ME sensor is magnetostrictive itself, the mechanical vibrations generate a magnetic flux density that can be detected wirelessly by a pickup coil without direct physical connections, and the sensor is entirely passive containing no internal power supply^21^. A network analyzer operating in the S_11_ mode, which is an ideal device to sense the resonance frequency, is used to apply an alternating voltage to the coil and monitor the flux changes-induced current changes in the coil. For a ribbon-like ME sensor of length *L*, density ρ, elastic modulus *E*, and Poisson’s ratio *v*, the fundamental resonance frequency *f*_0_ is given by equation (1)^22^.

1 $${f}_{0}=\frac{1}{2L}\sqrt{\frac{E}{\rho (1-{v}^{2})}}$$

A small extra mass load Δ*m* deposited on the sensor of mass M (${\rm{\Delta }}m\ll M$), the shift in the resonance frequency (Δ*f*) is described by equation (2)^23^.

2 $$\frac{{\rm{\Delta }}f}{{\rm{\Delta }}m}=-\frac{{f}_{0}}{2M}$$

From equation (2), we can conclude that an extra mass load on the sensor surface leads to a decrease of *f*_0_. As the resonance frequency is sensitive to the mass change, the sensor modified with a chemically or biologically sensitive polymer can be designed for detection of several bacteria and protein, such as *Bacillus anthracis spores* ^24,25^, *Salmonella typhimurium* ^26^, octachlorostyrene^27^. As well known, signal amplification is a critical challenge to the biological detection. In order to improve the sensitivity of the ME sensor, a sandwich enzyme-linked immunoassay to amplify mass signal has been widely investigated^28,29^.

In this study, we design a wireless, low-cost, high-sensitive and disposable ME biosensor for the detection of CSFV E2 antibody based on antigen-antibody reaction using an enzyme catalytic precipitation scheme to amplify the signal. The sensing configuration involves a sandwich immunoassay, in which E2 glycoprotein was immobilized on the gold-coated ME sensor surface and alkaline phosphatase (AP) conjugated goat anti-rabbit IgG was employed as a secondary antibody. The sandwich immunoassay formed on the ME sensor surface were detected by enzymatically converting 5-bromo-4-chloro-3-indolyl phosphate/nitro blue tetrazolium chloride (BCIP/NBT substrate) into an insoluble product deposited on the ME biosensor surface, consequently changing its resonance frequency which are correlated with the amount of CSFV E2 antibody.

## Materials and Methods

### Materials

CSFV E2 glycoprotein, rabbit anti-CSFV E2 antibody, AP-conjugated goat anti-rabbit IgG and BCIP/NBT substrate were obtained from Beijing Bo Sheng Bio-technology Co., Ltd. Anti-PRV (porcine pseudorabies virus) antibody, anti-PCV2 (porcine circovirus type2) antibody and fluorescein isothiocyanate (FITC)-labeled anti-CSFV E2 antibody were acquired from Green biological technology corporation, Ltd., China. 11-Mercaptoundecanoic acid, 1-ethyl-3-(3-dimethylaminopropyl) carbodimide hydrochloride (EDC), N-hydroxysulfosuccinimide (NHS), bovine serum albumin (BSA, 99%) and phosphate buffered saline (PBS buffer, pH = 7.4) were purchased from Sigma-Aldrich Corporation (Saint Louis, MO, USA).

### ME biosensor fabrication

#### Preparation of ME sensor platform

The ME sensor platforms composed of Metglas alloy 2826 (Fe_40_Ni_40_P_14_B_6_) were purchased from Honeywell Corporation (Morristown, NJ, USA). The sensor platforms with dimensions of 5 mm × 1 mm × 28 μm, were cut from a ribbon with size of 37 mm × 6 mm ×28 μm using a computer-controlled laser cutter. After laser cutting, to remove debris and organic film, the sensor platforms were ultrasonically cleaned in ethanol for 20 min and rinsed in deionized water, then dried in a stream of nitrogen. In order to protect the sensors from corrosion and improve the bio-compatibility of the sensor surface for E2 glycoprotein immobilization, both sides of the cleaned sensor platforms were sputtered with chromium (100 nm thick), followed by gold (100 nm thick). Then, the gold-coated sensor platforms were annealed in a vacuum oven at 200 °C for 3 h to relieve residual internal stress and promote the adhesion of the gold film to the ME ribbon. The annealed gold-coated sensor platforms were ready for E2 glycoprotein immobilization to form the ME biosensor.

#### Functionalization of the sensor surface

**Figure 1:**
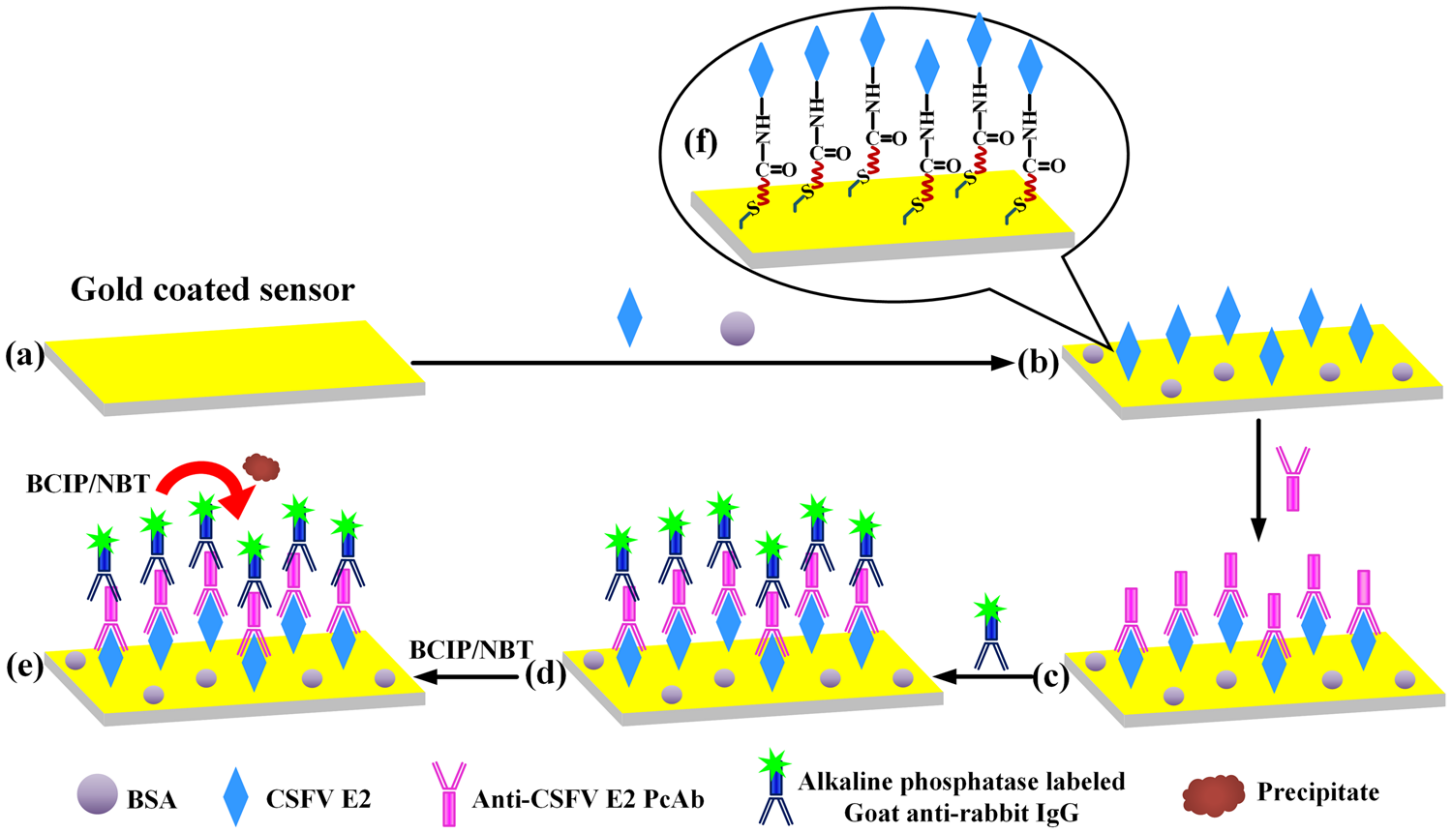
Schematic representation of the procedures of the ME biosensors functionalization.

The gold-coated sensor platforms (Fig. 1a) were ultrasonically cleaned with acetone, isopropanol, deionized water and ethanol for 10 min each, and dried under a stream of nitrogen. Cleaned sensor platforms were then immersed in a solution of 10 mM 11-mercaptoundecanoic acid in ethanol overnight at room temperature. After reaction, the SAM-modified sensors were rinsed several times with ethanol and distilled water, then dried under a stream of nitrogen. To activate the terminal carboxylic groups to the NHS ester, the sensors were dipped into a solution containing 40 mM EDC and 10 mM NHS in distilled water for 1 h, rinsed with deionized water, and dried under nitrogen. Subsequently, the activated sensors were incubated in 200 μL CSFV E2 with a certain concentration for 1 h at 37 °C, upon which the sensors were rinsed with PBS. To prevent non-specific adsorption, the E2-coated sensors were treated with 0.5% BSA for 20 min and rinsed with PBS to remove the excess BSA, as shown in Fig. 1b. Then, the sensors were incubated with 50 μL of PBS spiked with different concentrations of rabbit anti-CSFV E2 antibody (from 5 ${\rm{ng}}/{\rm{mL}}$ to 10 ${\rm{\mu }}{\rm{g}}/{\rm{mL}}$) for 1 h at 37 °C, as depicted in Fig. 1c. After rinsing with PBS buffer, the sensors were immersed into 50 μL of AP-conjugated goat anti-rabbit IgG (diluted 1:400 with PBS) for 1 h at 37 °C. Afterwards, the sensors were thoroughly washed with PBS buffer and deionized water to remove any nonspecifically bound AP-conjugated goat anti-rabbit IgG. Finally, the ME biosensors were fabricated, as shown in Fig. 1d. As a reference, supplementary Fig. S1 shows the real picture of the sensor platform before and after fabrication.

#### Optimization of the concentration of CSFV E2 immobilization

As the performance of the ME biosensor is greatly influenced by the density and distribution of CSFV E2 as the sensing membrane on the sensor surface, the working concentration of coating CSFV E2 is an important factor to optimize. The biosensors coated with CSFV E2 at different concentrations of 10 ${\rm{\mu }}{\rm{g}}/{\rm{mL}}$, 30 ${\rm{\mu }}{\rm{g}}/{\rm{mL}}$ and 50 ${\rm{\mu }}{\rm{g}}/{\rm{mL}}$ were incubated with FITC-labeled anti-CSFV E2 antibody at the identical conditions for 1 h at 37 °C. After reaction, the biosensors were rinsed several times with distilled water and dried under a stream of nitrogen, and then observed with a fluorescence microscope (DM 3000, Leica Microsystems Ltd.; Wetzlar, Germany).

### Signal measurement

**Figure 2:**
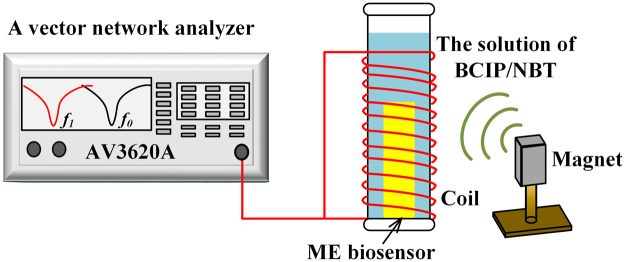
The schematic representation of wireless ME biosensor measurement system.

An experimental setup made of a bench-top vector network analyzer (AV3620A, the 41^st^ Institute of CETC, Qingdao, China) connected with a coil wound around a glass tube was used to wirelessly measure the resonance frequency of the ME biosensor^30^, as schematically represented in Fig. 2. To generate an AC field, the network analyzer was operated in the S_11_ mode for providing a swept frequency signal to the coil and it can monitor the reflected signal from the coil. In addition, a DC field generated by a bar magnet was applied to enhance the resonance behavior. The biosensors, bound with different amounts of AP-conjugated goat anti-rabbit IgG induced with different concentrations of anti-CSFV E2 antibody, were vertically and wirelessly (without any wire connections with measurement system) inserted into the glass tube containing 40 μL of BCIP/NBT substrate solution (pH = 9.5) sequentially, as shown in Fig. 1e. At each concentration, the resonance frequency of the biosensor was monitored and recorded every 5 min.

## Results and Discussion

### Assessment for the modification and detection process of the biosensor surface

#### SEM and EDS analysis

**Figure 3:**
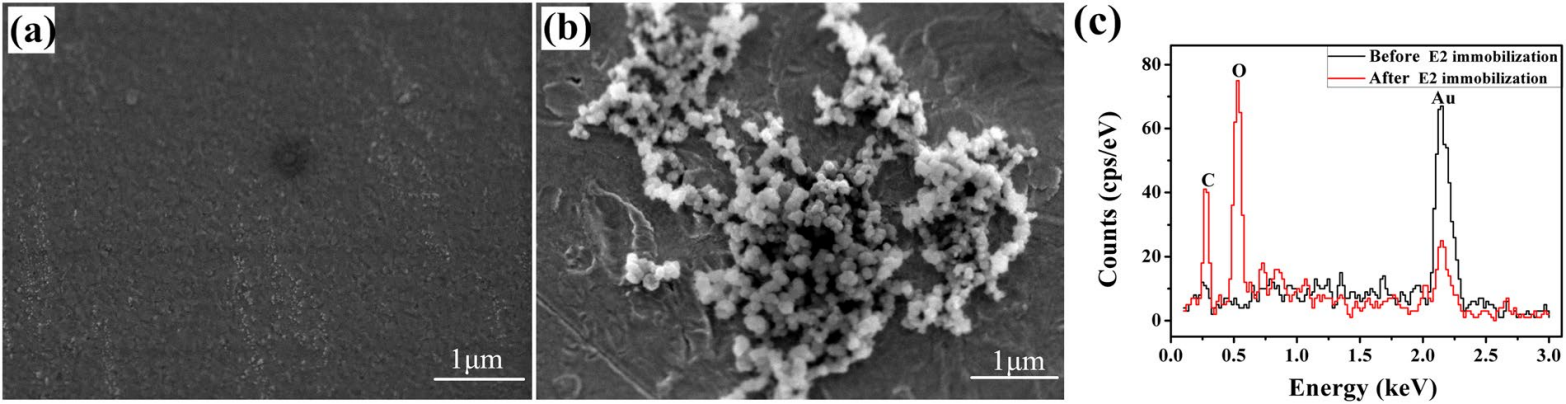
(**a**) SEM image of the gold-coated sensor surface. (**b**) SEM image of biosensor surface after CSFV E2 immobilization. (**c**) EDS spectrum of the biosensor surface before and after the CSFV E2 immobilization.

To confirm that CSFV E2 is immobilized on the biosensor surface, scanning electron microscopy (SEM) analysis and energy-dispersive spectroscopy (EDS) analysis were used to assess the procedure. The SEM examination was performed at 5 kV accelerating voltage using a JSM-7100F SEM (JEOL corporation, Tokyo, Japan). Figure 3a shows the SEM image of the ME biosensor gold surface without functionalization. It can be seen that the surface is smooth and naked. After the biosensor surface is modified with CSFV E2, spherical aggregates were presented on the surface, as observed in Fig. 3b. The schematic diagram of modification process is depicted in Fig. 1f. The formation of the carboxylate-terminated SAM on a gold-coated sensor is based on the chemisorption of the sulfur atom onto the metal surface (Au-S) through a metal-thiolate bond^31^. Then EDC/NHS solution provides a suitable and stable linker compounds that form a strong link between CSFV E2 and SAM. EDC is utilized as a coupling agent to activate carboxyl groups, and NHS provides a stable NHS ester. Thus, CSFV E2 is covalently linked to SAM through amide bond (-CONH-) corresponding to the replacement of NHS ester by CSFV E2 to form a peptide linkage^32^. Furthermore, the EDS spectrums for elemental analysis of the biosensor surface before and after the immobilization of CSFV E2 are compared in Fig. 3c. It is evident that the accumulation of carbon and oxygen increase dramatically, while the content of gold decrease after the CSFV E2 immobilization. E2 contains large amounts of carbon and oxygen due to its essence of envelope glycoprotein. Given the above analysis, it can be concluded that CSFV E2 is immobilized on the biosensor surface successfully.

#### Fluorescence microscopy analysis

**Figure 4:**
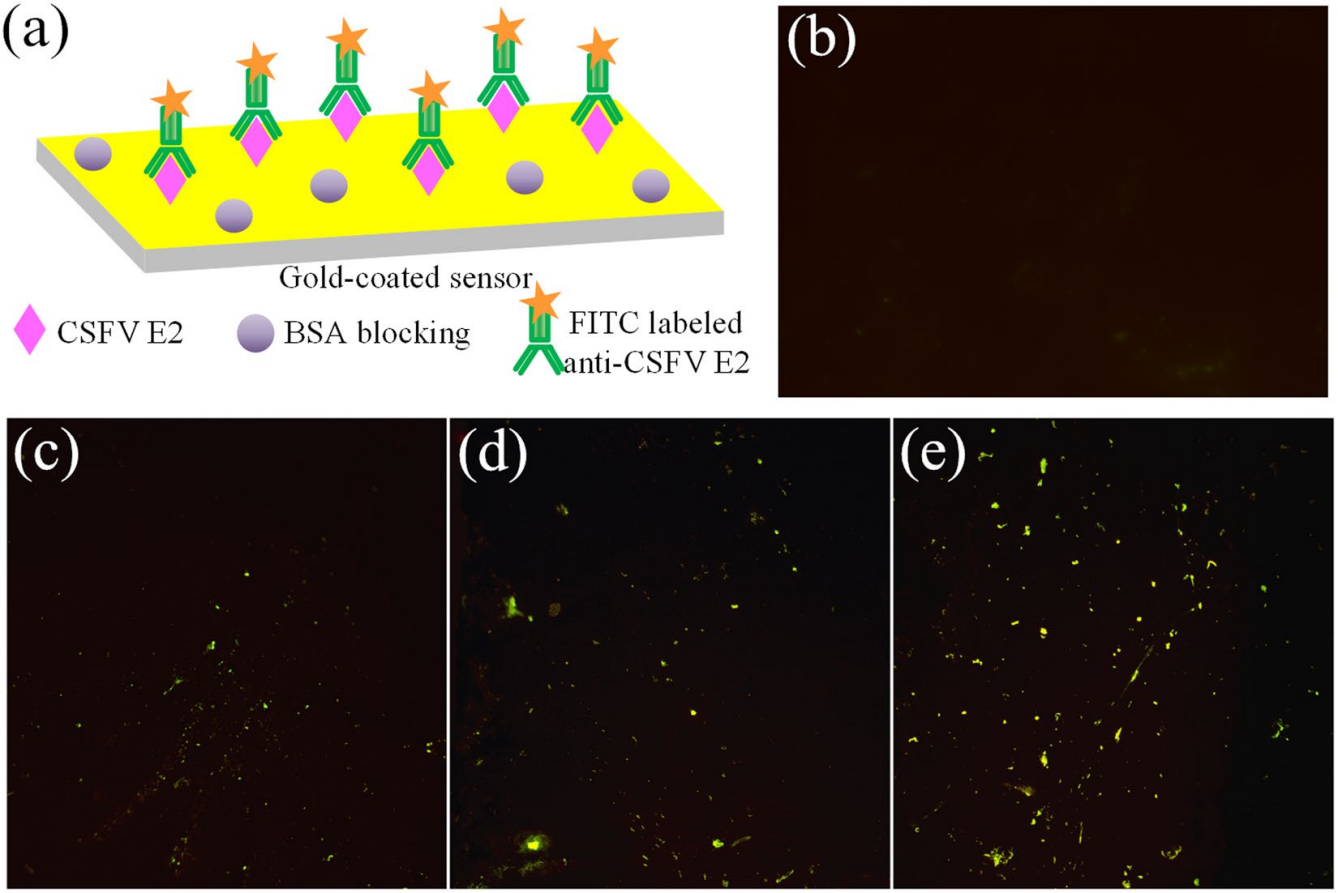
(**a**) Schematic diagram of FITC-labeled anti-CSFV E2 antibody captured by CSFV E2 modified on the biosensor surface. Fluorescence images of biosensors coated with CSFV E2 at different concentrations, including (**b**) 0 ${\rm{\mu }}{\rm{g}}/{\rm{mL}}$, (**c**) 10 ${\rm{\mu }}{\rm{g}}/{\rm{mL}}$, (**d**) 30 ${\rm{\mu }}{\rm{g}}/{\rm{mL}}$, (e) 50 ${\rm{\mu }}{\rm{g}}/{\rm{mL}}$.

Fluorescence microscopy was employed to evaluate the modification and detection processes of the biosensor surface. Fluorescein isothiocyanate (FITC) has the ability to react with amino groups and can be observed using a fluorescence microscope, thereby staining with FITC can be applied to confirm the presence of proteins^33,34^. Figure 4(c–e) depicts the fluorescence microscope images of the biosensors coated with CSFV E2 at different concentrations of 10 ${\rm{\mu }}g/{\rm{mL}}$, 30 ${\rm{\mu }}g/{\rm{mL}}$ and 50 ${\rm{\mu }}g/{\rm{mL}}$, respectively. Obviously, numerous green fluoresce spots of FITC-labeled anti-CSFV E2 molecules were observed on the biosensor surface owing to its combination with CSFV E2 modified on the surface, as schematically presented in Fig. 4a. As shown in Fig. 4(c–e), the number of green fluoresce spots increased with increasing concentrations of CSFV E2, and covered the whole area uniformly in Fig. 4e. Previous study have reported that Li *et al*. used 0.5 ${\rm{\mu }}{\rm{g}}/{\rm{mL}}$ as the coated concentration of CSFV E2 antigen^15^. Considering that the high concentration of CSFV E2 resulted in a high density of CSFV E2 on the sensor surface, leaving very little space for anti-CSFV E2 to bind^35^, 50 ${\rm{\mu }}{\rm{g}}/{\rm{mL}}$was selected as the optimal concentration for CSFV E2 immobilization. By contrast, the same experiment was carried out on a reference biosensor without CSFV E2 modification, as observed in Fig. 4b. There are almost no green fluorescence spots compared with the CSFV E2-coated biosensor under identical conditions, namely, there is no anti-CSFV E2 antibody absorbed to the biosensor surface. The results clearly clarify that CSFV E2 has been immobilized on the biosensor surface and additionally demonstrate that anti-CSFV E2 antibody is successfully captured by CSFV E2.

### Application of ME biosensors for anti-CSFV E2 antibody detection

**Figure 5:**
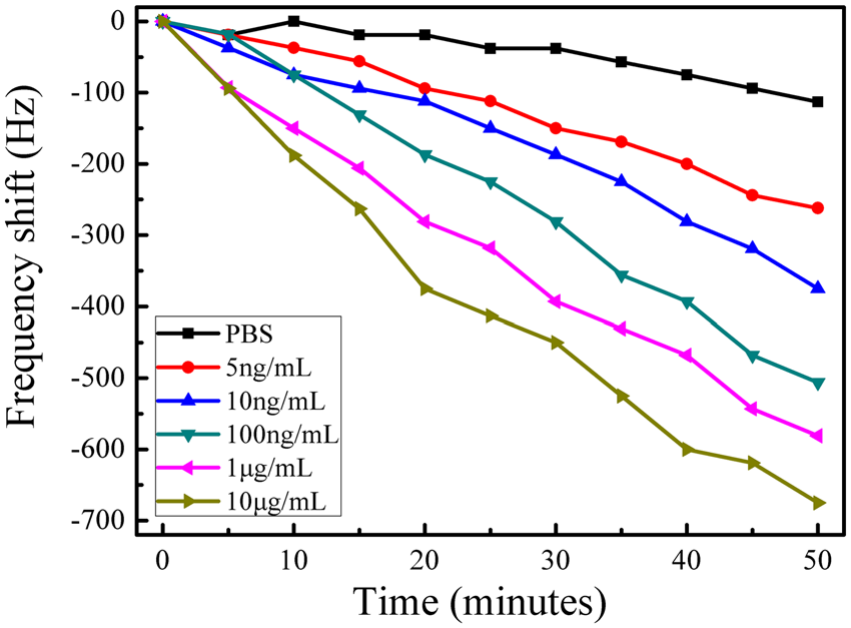
Time-dependent frequency responses at different anti-CSFV E2 antibody concentrations ranging from 0 to 10 ${\rm{\mu }}{\rm{g}}/{\rm{mL}}$.

Figure 5 depicts the dynamic response to the enzymatic catalytic reaction on wireless ME biosensors surface with anti-CSFV E2 antibody concentration ranging from 0 to 10 ${\rm{\mu }}{\rm{g}}/{\rm{mL}}$ as a function of immersion time. When anti-CSFV E2 antibody in the sample solution bound with CSFV E2 on the surface, the subsequent binding of AP- conjugated goat anti-rabbit IgG and rabbit anti-CSFV E2 on the surface formed a sandwich complex, as shown in Fig. 1d. By the catalysis of alkaline phosphatase, BCIP was hydrolyzed to yield a product that reacted with NBT to form insoluble blue NBT-formazan^36^, as chemically represented in supplementary Fig. S2. The produced precipitate was strongly bound to the biosensor surface, as illustrated in Fig. 1e, which in turn caused a decrease in its resonance frequency after an induction period, as shown in Fig. 5. This induction period is attributed to the time required for the BCIP/NBT precipitate to adhere to the biosensor surface. Steady-state response is generally achieved at about 50 min for 5 ${\rm{ng}}/{\rm{mL}}$ anti-CSFV E2 or less. Biosensors exposed to higher concentrations of anti-CSFV E2 require longer time to attain stable state. It is evident from Fig. 5 that the rate and magnitude of resonance frequency shift increase with increasing anti-CSFV E2 concentrations. Since conjugate enzyme AP is introduced onto the biosensor surface based on the specific binding of the CSFV E2 and anti-CSFV E2 in the sample and sequential combination of anti-CSFV E2 and AP-conjugated goat anti-rabbit IgG, AP is only present when anti-CSFV E2 is bound to the surface. In the detection scheme, biocatalyzed precipitation takes place only if AP, namely, the target anti-CSFV E2 is present in the sample except for the non-specific adsorption, so the amount of precipitation adhered to the surface quantitatively correlates with the anti-CSFV E2 concentration, corresponding to the changes in resonance frequency. In this way, the ME biosensor can be used to detect CSFV E2 antibody wirelessly.

The 0 ${\rm{\mu }}g/{\rm{mL}}$ curve of Fig. 5 represents a background response of the blank control sensor which is processed through the same series of experimental procedures (without anti-CSFV E2 antibody). A noise level of 90 Hz is observed due to the non-specific binding of AP- conjugated goat anti-rabbit IgG. However, there is a 262-Hz change in resonance frequency at the 5 ${\rm{ng}}/{\rm{mL}}$ anti-CSFV E2 concentration over the same time period, indicating that the non-specific adsorption can be ignored. Thus it is demonstrated that the response is only due to the attachment of CSFV E2 antibody to the biosensor surface.

**Figure 6:**
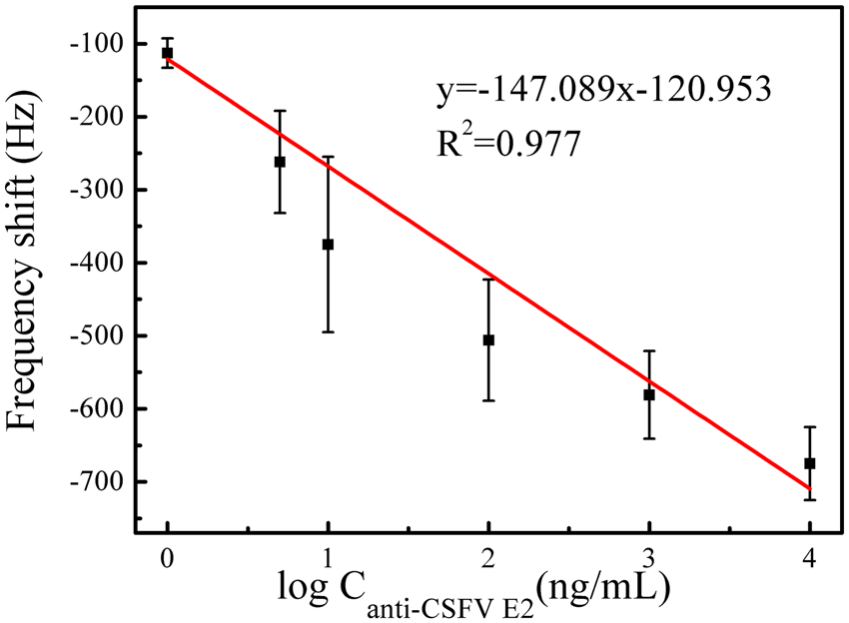
Calibration curve: the 50 min shift in resonance frequency as a function of different anti-CSFV E2 antibody concentrations.

Figure 6 shows the standard calibration curve corresponding to the 50 min change in resonance frequency versus the logarithmic value of the anti-CSFV E2 antibody concentrations ranging from 5 ${\rm{ng}}/{\rm{mL}}$ to 10 ${\rm{\mu }}{\rm{g}}/{\rm{mL}}.$ At each concentration, the biosensor calibration experiments were repeated five times or more under the identical conditions. In addition, the t-test was conducted for different concentrations at a 95% confidence interval. The calculated result of $p < 0.05$ showed that the change in frequency was statistically different for different concentrations. The biosensor response is linear in the range of 5 ${\rm{ng}}/{\rm{mL}}$ to 10 $\,{\rm{\mu }}{\rm{g}}/{\rm{mL}}$, with the sensitivity of 56.2 ${\rm{Hz}}/{\rm{\mu }}{\rm{g}}\cdot {m}{{L}}^{-1}$. The linear equation could be represented by ${\rm{\Delta }}f=-147.089\,\mathrm{log}\,{C}_{anti-CSFVE2}-120.953({R}^{2}=0.977)$. The limit of detection (LOD) is calculated to be 2.466 ${\rm{ng}}/{\rm{mL}}$, according to the equation (3)^37^.

3 $${\rm{LOD}}=3{S}_{B}/b$$

where *S*_*B*_ is the standard deviation of the blank sensor and *b* is the slope of the analytical curve, as shown in Fig. 6. The detection limit of our biosensor is significantly lower than that obtained with the SPR method of 10 $${\rm{ng}}/{\rm{mL}}$$ ^16^. Li *et al*. have demonstrated that the ME biosensor has a higher sensitivity than the piezoelectric microcantilever and other acoustic wave (AW) devices^38^. Besides, minimizing the ME biosensor size down to 5 mm × 1mm enhanced its sensitivity and cost-effectiveness compared with the previous study^39^. In addition, the ME biosensor method is relatively cost-effective due to no need of costly instruments and DNA purification kits, instead requiring only US$ 0.001 per sensor (US$ 500/kg of the ME material) and simple instrumentation. Besides, the ME biosensor method is comparatively fast, requiring only several minutes whereas TaqMan-based quantitative real-time PCR (qPCR) requires 2–3 h, and its operation principle is relatively easy^40^. Furthermore, previous researches^19,27,40^ have also demonstrated the significant advantages in terms of sensitivity, cost and ease of use of the ME biosensor methodology. For the detection of CSFV E2 antibody, various published methods are summarized in Table 1, showing the superior performance of our biosensor. Thus, compared with the conventional methods, the ME biosensor appears to be a fast, cost-effective, and simple tool in the detection for CSFV E2 antibody.

**Table 1:** Comparisons of performances between various methods for CSFV E2 antibody detection.

Methods	Sensitivity/ Detection limit	Cost	Ease of use	References
ME biosensor	56.2 $${\rm{Hz}}/{\rm{\mu }}g\cdot m{L}^{-1}$$; 2.466 $${\rm{ng}}/{\rm{mL}}$$	US$ 0.001/sensor; several minutes	Minimum skill; smaller size	This work
Surface plasmon resonance (SPR)	10 $${\rm{ng}}/{\rm{mL}}$$	1.5 hr	Needs skill	16
ELISA	100 $${\rm{ng}}/{\rm{mL}}$$	Time-consuming	Labor-intensive	16
Neutralizing assay		Time-consuming	Well set up cell culture laboratory	14
Single dilution immunoassay		Costly purification procedures	Work-intensive	14

### ME biosensor specificity

**Figure 7:**
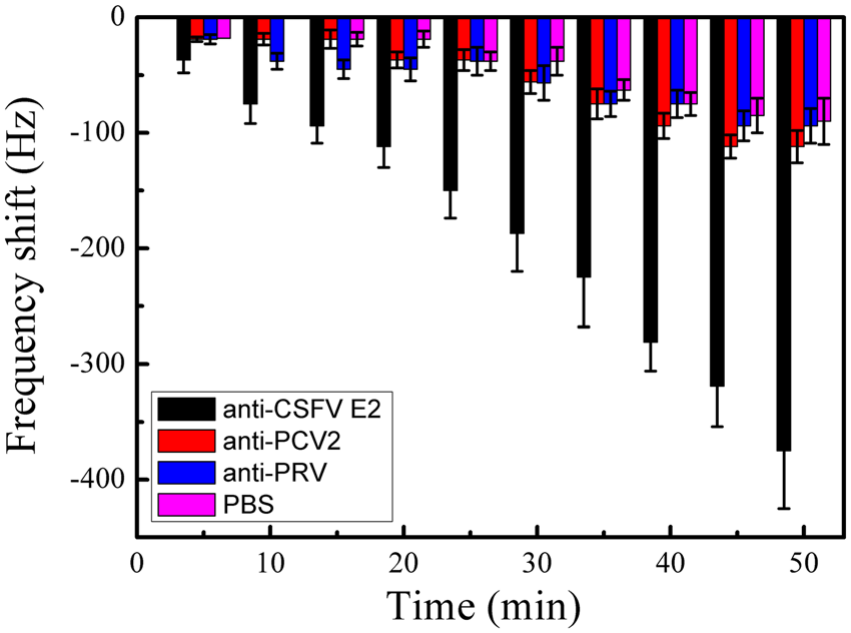
Real-time response of the ME biosensor to other interferents with the concentration of 10 ${\rm{ng}}/{\rm{mL}}$.

The ME biosensor specificity was investigated by determining the biosensor responses to anti-PRV antibody and anti-PCV2 antibody with a molecular structure similar to anti-CSFV E2, each at a concentration of 10 ${\rm{ng}}/{\rm{mL}}$. As shown in Fig. 7, the biosensor showed only insignificant response to these selected interferences due to non-specific absorption, with response levels similar to the blank sample PBS. The results demonstrate that the resonance frequency shift is only due to the specific binding of the anti-CSFV E2 antibody and CSFV E2 modified on the biosensor surface. In addition, the ME biosensor shows strong specific binding to CSFV E2 antibody, demonstrating the potential feasibility of its application in real serum samples measurement.

## Conclusions

For the first time, a wireless ME biosensor was designed for the detection of CSFV E2 antibody based on antibody-antigen binding with enzymatic catalytic signal amplification. The ME biosensor coupled with formation of a sandwich antigen–antibody complex by CSFV E2 immobilized on the ME sensor surface, the target rabbit anti-CSFV E2 antibody and AP-labeled secondary goat anti-rabbit IgG was demonstrated. Alkalin–e phosphatase as a labeled enzyme catalyzes BCIP/NBT substrate to produce precipitation adhered to the biosensor surface, resulting in enhanced mass loading that leads to a shift in resonance frequency correlating with the CSFV E2 antibody concentration. The modification and detection process were confirmed by SEM, EDS and fluorescence microscopy analysis. A linear relationship was found between the resonance frequency shift and the logarithm of CSFV E2 antibody concentrations ranging from 5 ${\rm{ng}}/{\rm{mL}}$ to 10 ${\rm{\mu }}{\rm{g}}/{\rm{mL}}$, with the detection limit of 2.466 ${\rm{ng}}/{\rm{mL}}$ and the sensitivity of 56.2 ${\rm{Hz}}/{\rm{\mu }}{\rm{g}}\cdot {\rm{m}}{L}^{-1}$, which is comparable with that reported in the previous research. Since this study focused on the first application of CSFV E2-coated ME biosensor as a new method for the anti-CSFV E2 antibody detection, the biosensor was applied to purified anti-CSFV E2 antibody instead of real serum samples. Future work will be performed by the direct application of biosensors in the serum samples for real life diagnostic purposes. The study not only proposed a new method for the detection of CSFV E2 antibody, but also indicated its potential practicability in the real life diagnose.

## Electronic supplementary material

Supplementary information
