# Supplementary material for: A magnetoelastic biosensor based on E2 glycoprotein for wireless detection of classical swine fever virus E2 antibody
Source: Sci Rep. 2017 Nov 15;7:15626. doi: 10.1038/s41598-017-15908-2 (PMC5688166; doi:10.1038/s41598-017-15908-2)
Supplement: Supplementary file 1 — Supplementary information [file 41598_2017_15908_MOESM1_ESM.pdf]

Supplementary information:

A magnetoelastic biosensor based on E2 glycoprotein for wireless detection of classical swine fever virus E2 antibody

Xing Guo[1]†, Shengbo Sang[1]†*, Jinyu Guo[1], Aoqun Jian[1], Qianqian Duan[1], Jianlong Ji[1], Qiang Zhang[1], Wendong Zhang[1]

[1]MicroNano System Research Center, Key Lab of Advanced Transducers and Intelligent Control System of the Ministry of Education & College of Information Engineering, Taiyuan University of Technology, Jinzhong 030600, China.

† These authors contributed equally to this work.

*Address correspondence to: sunboa-sang@tyut.edu.cn (Shengbo Sang)

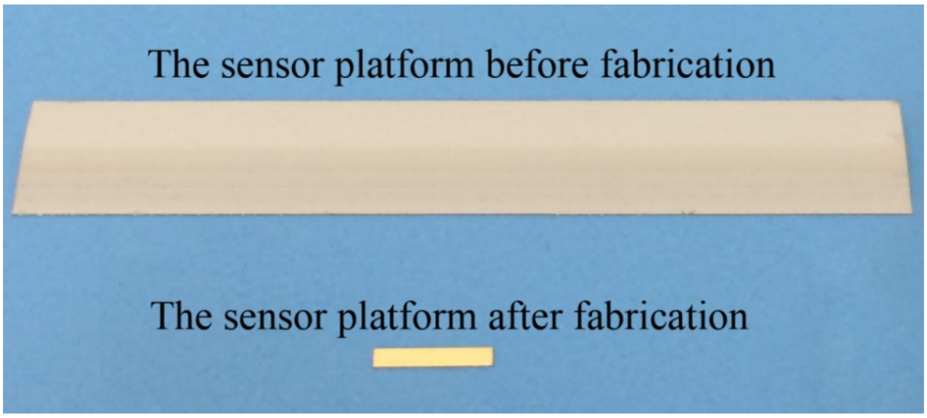

Fig. S1: The real picture of the sensor platform before and after fabrication.

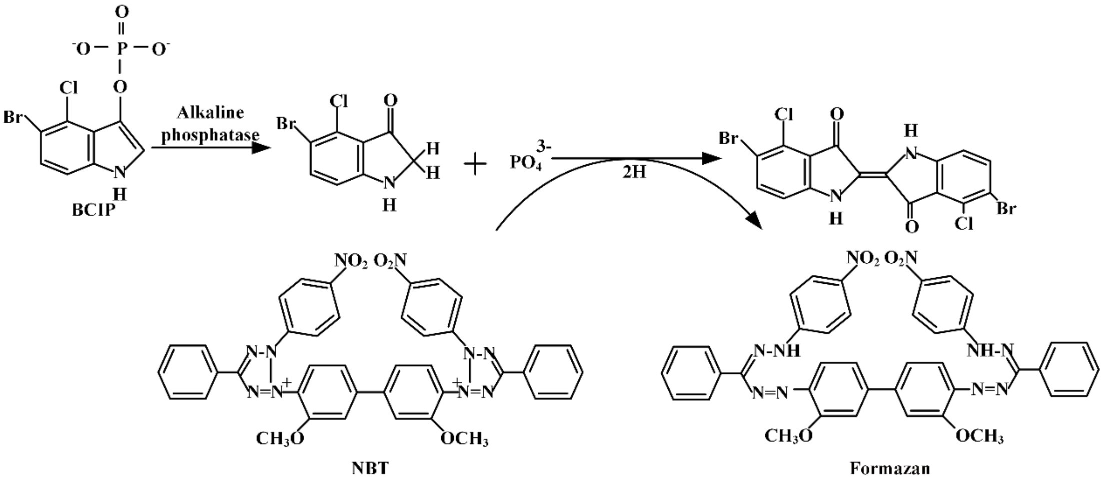

Fig. S2. Chemical equations of catalytic BCIP/NBT precipitation reaction mechanism.
